# Changes in Symptom Networks During Inpatient Cancer Rehabilitation: A Retrospective Bayesian Gaussian Graphical Model Analysis of Real-World Patient-Reported Outcomes

**DOI:** 10.3390/cancers18132155

**Published:** 2026-07-04

**Authors:** Christina Kirchhoff, Thomas Licht, Samuel Eke, Špela Matko, Vincent Grote, Michael J. Fischer, Katharina Hüfner, David Riedl

**Affiliations:** 1 Department of Psychiatry, Psychotherapy, Psychosomatics and Medical Psychology, University Hospital of Psychiatry II, Medical University of Innsbruck, 6020 Innsbruck, Austria; 2 Ludwig Boltzman Institute for Rehabilitation Research, Ludwig Boltzmann Society, 1140 Vienna, Austria; 3 Oncological Rehabilitation Center Sankt Veit im Pongau, 5621 St.Veit im Pongau, Austria; 4 Department of Orthopedics and Traumatology, Medical University Graz, 8010 Graz, Austria; 5 VITREA Rehabilitation Center Kitzbühel, 6370 Kitzbühel, Austria

**Keywords:** network analysis, Bayesian networks, Gaussian Graphical Models, outcome, structural change, cancer survivorship, depression, anxiety

## Abstract

**Background/Objectives:** Cancer survivors admitted to inpatient rehabilitation suffer from a complex burden of interrelated physical and psychological symptoms. While mean-level improvements during rehabilitation are well-documented, it remains unknown whether rehabilitation modifies the underlying structure of symptom interconnections—the symptom network—beyond reducing individual symptom scores. This study aimed to characterize symptom network structure at admission and discharge of a 21-day inpatient cancer rehabilitation program based on cancer-related physical symptoms and psychosocial functioning, formally compare network topology across timepoints, identify structurally central treatment targets, and assess the transdiagnostic generalizability of findings. **Methods:** Secondary analysis of routinely collected, electronic patient-reported outcome (PRO) data from 5066 cancer survivors (mean age 60.3 years, SD 12.2; 64.2% female; most frequent diagnoses: breast cancer = 36.9%, hematological malignancies = 10.4%; prostate cancer = 8.5%) admitted to a single-center inpatient rehabilitation program was performed between January 2017 and November 2022. The EORTC QLQ-C30 and the Hospital Anxiety and Depression Scale (HADS) questionnaires were utilized. Bayesian Gaussian Graphical Models were estimated at admission (T0) and discharge (T1) across 17 symptom and functioning domains using Bayesian Model Averaging (15,000 iterations). Edge-level change was quantified via posterior distributions of pairwise differences with 95% Highest Density Intervals. Node-level changes were assessed using Bayesian paired t-tests. Centrality was quantified by Expected Influence and Bridge Expected Influence. **Results:** Patients showed clinically meaningful improvements across all 17 domains during rehabilitation (all Bayes Factors >10; posterior probability of direction >99.9%). The largest standardized effects were observed for emotional functioning (Cohen’s d = 0.76), global health status (d = 0.69), and fatigue (d = 0.53). These improvements were clinically meaningful for a substantial proportion of patients: 62% improved by at least the minimal important difference in fatigue and 58% in emotional functioning, and the proportion of patients with probable anxiety fell from 15% to 6% and probable depression from 10% to 4%. Emotional functioning and anxiety were the most central domains in the symptom network—most strongly connected to the rest of patients’ symptom burden—at both admission and discharge. Despite the clinical improvements, the overall architecture of symptom interconnections changed little (83% of connections were unchanged). This indicates that the severity of symptoms was mitigated while the structure linking them together remained largely intact. The one connection that strengthened was that between impaired social functioning and financial difficulties (Δ = −0.112). Structural findings were consistent across ten cancer types (leave-one-out r > 0.80 in seven of ten). **Conclusions:** Over the course of inpatient cancer rehabilitation, patients showed large improvements against a background of largely stable symptom network architecture. Emotional functioning and anxiety occupy structurally central positions at both admission and discharge, identifying them as candidate domains warranting further investigation for network-informed rehabilitation. These findings provide a novel structural perspective on oncological rehabilitation and a framework for developing more targeted intervention strategies.

## Simple Summary

Cancer survivors who undergo inpatient rehabilitation experience many physical and psychological symptoms that are interconnected. Rather than studying symptoms one at a time, we mapped how 17 symptom and functioning domains relate to one another, and how those relationships change over a three-week inpatient program in 5066 survivors. Patients improved across all domains, with the largest gains observed in emotional functioning and fatigue, and far fewer patients had probable anxiety or depression at discharge than at admission. At the same time, the overall structure of symptom connections remained largely stable during rehabilitation, suggesting that symptom interconnections are a robust feature of the cancer survivorship experience. Importantly, emotional functioning and anxiety emerged as the most highly connected nodes in the network, suggesting that emotional functioning and anxiety may be clinically relevant domains for further investigation as candidate domains for future intervention studies.

## 1. Introduction

In recent decades, advances in oncological treatment have led to substantially improved survival rates across a wide range of cancer diagnoses, which resulted in a growing population of cancer survivors with lasting physical and psychological side effects from both disease and treatment [1,2]. The most commonly reported burdens are fatigue, pain, sleep disturbance, anxiety, depression, and impaired physical and social functioning [3,4,5,6,7]. A biopsychosocial perspective is essential to comprehensively understand the health status of cancer survivors, as physical, psychological, and social symptoms are closely interrelated and may reinforce one another over time [8,9]. These symptoms may form dynamic feedback cycles, in which individual symptoms simultaneously act as both consequences and maintaining factors of the overall symptom burden [10]. Such complex relationships have been described across multiple survivorship-related symptoms [11]. For example, depression has been linked to pain, fatigue and anxiety, suggesting that symptom burden may arise from interacting processes rather than isolated effects [3,12].

To better understand these dynamic interacting symptom processes and their potential relevance for rehabilitation, analytical approaches are needed that capture the multidimensional structure of symptom burden in cancer survivorship. One increasingly used approach is network analysis, which conceptualizes symptoms and functioning domains as mutually connected components within a system rather than as independent outcomes or reflections of a single underlying construct [13,14,15]. To date, network analysis has been used primarily in psychology, particularly for the analysis of complex or multivariate data [5,6].

One strategy to investigate the structure and simultaneous occurrence of symptoms and functioning in cancer patients is network analysis. In these models, symptoms and functioning domains are visually represented as ‘nodes’, and the connections between them are represented as ‘edges’. A connection indicates that two domains remain associated even after all other symptoms in the model have been taken into account [13,14,15]. In contrast to traditional analyses that examine each symptom separately or summarize several symptoms into a total score, network analysis focuses on the pattern of associations among symptoms. In other words, this approach can identify domains that are especially strongly connected to the rest of the symptom system and may therefore be clinically relevant treatment targets. Thus, network analyses make it possible to examine symptom co-occurrence that moves beyond traditional sum-score approaches. Network analyses specifically offer two advantages over traditional approaches: for one, it is possible to identify symptoms that are densely connected to many other symptoms, which is defined as *high centrality*, to identify important targets for interventions (for example, fatigue may be closely linked to reduced physical functioning, emotional distress, and reduced social participation). The centrality of variables is often quantified by calculation of their expected influence. To calculate the expected influence, the strength of all connections a symptom has with others in the network is summed up, with higher values indicating symptoms that are more influential within the overall symptom system. It is assumed that improvement in a symptom with high centrality would indirectly influence the whole network with all associated symptoms [16]. Additionally, some symptoms may also serve as *bridge nodes*, which means that they connect distinct symptom clusters, such as somatic symptoms and psychological symptoms. Improvement in these bridge node symptoms would then lead to symptom spillover across domains (e.g., improvement in mood would lead to a healthier and more active lifestyle) [17]. To evaluate the stability and reliability of these connections in the network, the edge inclusion probability (PIP) can be calculated. The PIP can be understood as the probability that a given connection is truly present in the network rather than reflecting chance. It ranges from 0 to 1, where values close to 1 mean there is strong evidence that two symptoms are genuinely connected, and values close to 0 mean the connection is unlikely to be real. Higher PIP values therefore indicate more trustworthy connections.

Cancer rehabilitation aims to improve the full range of physical and psychological impairment caused by the malignant disease and its treatments through multimodal, interdisciplinary treatment [18,19]. Several studies have shown substantial symptom improvement during inpatient cancer rehabilitation [20,21,22,23]. However, so far only very limited knowledge is available about whether or not the underlying structure of how symptoms are interrelated—i.e., the symptom network—also changes during inpatient rehabilitation, which would indicate network-level changes beyond symptom reduction. From a clinical point of view, this is thought to be highly relevant, since structural stability would indicate that symptom interconnections are stable features of the oncological disease experience independent of treatment intensity. Conversely, structural reorganization would suggest that rehabilitation would be consistent with a loosening of maladaptive symptom coupling. While network analyses are increasingly used in cancer research to deepen the understanding of symptom interconnectivity and improve the effectiveness of interventions, this is still a relatively young and growing field of research, and there is a lack of high-quality long-term studies [24]. Consequently, it remains unclear to what extent symptom network structures are consistent across different cancer diagnoses and therefore reflect transdiagnostic patterns of survivorship burden.

The aim of this study was to investigate networks of symptoms and functioning domains in a large and diagnostically heterogeneous sample of cancer survivors undergoing a three-week multimodal inpatient cancer rehabilitation program. Bayesian Gaussian Graphical Models were chosen because they directly answer the question of which connections between symptoms are likely to be present, and to what extent those connections plausibly change between admission and discharge of the rehabilitation. In simpler terms, the Bayesian model does not only give a single estimated connection between two domains. It also gives an estimate of how confident we can be that this connection exists. Unlike a simple correlation analysis, the estimated connections represent relationships between two symptom domains after controlling for all other variables in the network. This allows the network to identify direct rather than indirect associations. By examining symptom networks both at admission and discharge, we aimed to characterize the structure of symptom burden at the beginning of rehabilitation and to explore potential changes in symptom interrelationships following treatment. In addition, we sought to identify highly central and bridge symptoms that may represent clinically relevant targets for rehabilitation interventions. Finally, to evaluate the robustness and generalizability of the findings, network structures were compared across ten diagnostic subgroups within the sample.

## 2. Materials and Methods

### 2.1. Study Design and Participants

This study is based on data collected in routine clinical care between January 2017 and November 2022 at the Oncological Rehabilitation Centre St. Veit im Pongau (Austria). The dataset and study procedures are described in detail in previous studies [20,21,25]. Briefly, adult cancer survivors underwent a 21-day multidisciplinary inpatient rehabilitation program which was covered by the Austrian pension funds. The rehabilitation program followed a standardized multidisciplinary approach and consisted of physical therapy, aerobic and resistance training, psycho-oncological counseling (individual and group), psychoeducational interventions, nutritional counseling, social counseling, relaxation therapies, and occupational therapy. The minimal therapeutic intake was 1800 min during the 21-day treatment. A detailed overview of the median number of treatment modalities during the rehabilitation measure has been reported before [20].

Patients completed electronic patient-reported outcomes (ePRO) using the Computer-based Health Evaluation Software (CHES) [26]. The baseline assessment (T0) was completed by patients at home prior to admission via a web-based patient portal. The discharge assessment (T1) was completed on tablets provided at the rehabilitation center during the final four days of the 21-day stay. The median interval between the baseline assessment and admission was 15 days (IQR 6–29), and that between the discharge assessment and actual discharge was 4 days (IQR 2–6). In cases of repeated admissions, only the first stay was included in this study to exclude potential bias. Furthermore, patients were excluded if they had (a) terminated rehabilitation within the first three days of treatment, (b) an interval exceeding 56 days between T0 assessment and admission, or (c) incomplete questionnaire data. Of the cancer survivors who met the predefined inclusion criteria during the study period (adults completing inpatient rehabilitation; first admission only in cases of repeated stays; an interval of no more than 56 days between the baseline assessment and admission; and no termination of rehabilitation within the first three days), the present analysis used a complete-case approach. Patients were retained only if they had complete data on all 17 network variables (15 EORTC QLQ-C30 scales and the two HADS subscales) at both admission (T0) and discharge (T1). Sociodemographic and clinical characteristics (age, sex, and grouped ICD-10 tumor type) were extracted for the resulting analytical sample and are reported descriptively.

### 2.2. Measures of Quality of Life, Symptoms, Functions and Psychological Distress

**EORTC QLQ-C30:** Health-related quality of life (HRQOL) and symptom burden were assessed using the EORTC QLQ-C30 questionnaire [7]. The EORTC QLQ-C30 consists of 30 items which can be scored into one global HRQOL scale, five functional scales (physical, role, emotional, cognitive, and social functioning), and nine symptom scales (fatigue, nausea/vomiting, pain, dyspnoea, insomnia, appetite loss, constipation, diarrhea, and financial impact). All scales were scored according to the EORTC scoring manual, transforming raw scores linearly to a 0–100 scale. For functional and global HRQOL scales, higher scores indicate better functioning, while for symptom scales, higher scores indicate greater burden.

**HADS:** Psychological distress was assessed with the Hospital Anxiety and Depression Scale (HADS) [27]. The HADS consists of 14 items which can be scored into an anxiety (referred to as HADS-A) and a depression (HADS-D) subscale. Both scales range between 0 and 21 points, with higher scores indicating greater distress, and scores ≥ 11 are typically interpreted to represent probable clinical caseness on the respective scale, while scores 7–10 indicate borderline caseness.

### 2.3. Statistical Analysis

All analyses used a complete-case approach: Only patients with complete data on all 17 network variables at both admission and discharge were included. To assess potential selection bias, included (*n* = 5066) and excluded (*n* = 505) patients were compared on age, sex, and tumor type. The EORTC QLQ-C30 scale and HADS were treated as continuous variables; the Gaussian copula estimator implemented in BGGM does not assume multivariate normality and is robust to departures from it. Multicollinearity was examined through the pairwise correlation matrix and variance inflation factors (VIF), with VIF values < 5 considered acceptable [28].

**Network estimation:** To estimate networks, Bayesian Gaussian Graphical Models (BGGMs) were calculated separately at T0 and T1 using Bayesian Model Averaging (BMA) as implemented in the easybgm package [29] with the BGGM backend [30] in R (2026.01.1 Build 403). The networks show how patients with higher scores on one domain tended to differ from other patients on another domain at the same timepoint, after accounting for the remaining domains. We chose a Bayesian approach rather than the more common frequentist (graphical-LASSO) method for three reasons. First, it gives a direct, intuitive measure of how confident we can be that each connection between symptoms is real (the Posterior Inclusion Probability). Second, it does not depend on an arbitrary tuning setting that can strongly influence which connections appear in frequentist networks. Third, it provides a full range of plausible values for each connection and for how much it changed between admission and discharge, which is what allowed us to formally compare the networks at the two timepoints. Each network included 17 nodes corresponding to the fifteen EORTC QLQ-C30 scales and two HADS subscales. To ensure reproducibility, all models were estimated with 15,000 posterior sampling iterations and a fixed random seed (2024).

Edges in BMA-based networks are represented as BMA-weighted partial correlation coefficients, where each edge weight reflects the posterior expectation across all possible network structures, weighted by their respective model probabilities. Edge inclusion is quantified by the Posterior Inclusion Probability (PIP), defined as the summed posterior probability across all models containing a given edge. PIPs range from 0 to 1, with values ≥ 0.50 indicating that inclusion is more probable than exclusion, and values ≥ 0.75 indicating strong evidence for inclusion [31]. While edges with BMA-weighted partial correlations |r| < 0.10 were suppressed for the visualization of networks, all edges were retained in inferential analyses. Node layout was derived from the T0 network using the Fruchterman–Reingold spring algorithm and held constant across both networks to enable visual comparison. Markov Chain Monte Carlo (MCMC) convergence was assessed via effective sample size (ESS) for each edge parameter prior to inference, and ESS > 1000 was required for all parameters [32]. These models represent between-person conditional association structures estimated separately at each timepoint; they do not capture within-person temporal dynamics.

**Formal network comparison:** To quantify changes during rehabilitation in edge structure, posterior distributions of pairwise edge differences (T0 − T1) were estimated using BGGM::estimate (15,000 iterations). For each of the 136 unique edges, the posterior mean difference and 95% Highest Density Interval (HDI) were computed using the coda package [33]. Because 136 edge-level differences were examined, all edge-level comparisons were treated as exploratory; we did not pre-specify individual edges. To characterize the edge changes conservatively, we report three complementary criteria: (i) whether the 95% and 99% highest-density intervals (HDIs) of the posterior difference excluded zero (a criterion of directional reliability); (ii) the posterior probability of direction (pd); and (iii) a region-of-practical-equivalence (ROPE) criterion of ±0.05 on the partial-correlation scale (a criterion of magnitude). An edge was considered to show a non-trivial change only if its 95% HDI fell entirely outside the ROPE. In other words, to reduce overinterpretation, we now summarize edge changes using several complementary criteria: whether the 95% interval excludes zero, whether the 99% interval also supports the direction, the posterior probability of direction, and whether the change is large enough to fall outside a small range considered practically negligible.

**Centrality analysis:** Expected Influence (EI) [16] was computed as the signed sum of BMA-weighted edge weights, providing a measure of each node’s net activating or inhibitory role; negative EI indicates a predominantly inverse association with network neighbors. Bridge Expected Influence (Bridge EI) [17] was computed to quantify each node’s connectivity across three predefined communities: functioning scales (physical, role, social, emotional, cognitive functioning, global QoL), symptom scales (fatigue, nausea/vomiting, pain, dyspnoea, insomnia, appetite loss, constipation, diarrhea, financial impact), and psychological scales (HADS anxiety and depression). The three communities were defined a priori following the conceptual structure of the applied instruments: the EORTC QLQ-C30 distinguishes functioning from symptom scales, and the HADS represents a separately validated psychological-distress dimension. Bridge Expected Influence is conditional on this community assignment. All centrality analyses used the networktools package [34]. Financial difficulties are classified as a single-item symptom scale within the EORTC QLQ-C30 and were assigned to the symptom community on that basis. Because it is conceptually distinct from somatic symptoms, we conducted two sensitivity analyses: reassigning it to the functioning/social community for the Bridge Expected Influence computation, and re-estimating the network with it removed entirely.

**Node-level change:** Bayesian paired t-tests were conducted for each of the 17 nodes using the BayesFactor package version 0.9.12.4.8 [35] under the default Cauchy prior (scale = √2/2). For each node, the Bayes Factor (BF_10_) and posterior median difference with 95% HDI were reported (T0 − T1 convention: negative values indicate improvement for functioning scales; positive values indicate symptom reduction for symptom scales). Given the exploratory nature of these node-level tests, BF_10_ > 10 is used as the threshold for strong evidence [36].

**Standardization and effect size calculation:** To allow a direct comparison of mean change during treatment across the EORTC QLQ-C30 (0–100 scale) and HADS subscales (0–21 scale), all changes were standardized by calculation of Cohen’s d as the absolute posterior median difference divided by the standard deviation at T0 (|Δ|/SD T0). This standardization leads to scale-free effect sizes which are directly comparable across all instruments. Following established conventions, Cohen’s d = 0.5 was used as the threshold for clinically meaningful effects, representing a medium effect size. Clinical relevance was evaluated using established minimal important differences (MIDs). For the EORTC QLQ-C30 (0–100 scales), we applied the evidence-based guidelines of Cocks et al. [37], using 5 points as a small but potentially relevant change and 10 points as a clearly clinically relevant change. For the HADS subscales, MIDs of 1.3 (anxiety) and 1.4 (depression) were used [38]. Due to the large sample size, MID-based interpretation rather than statistical evidence alone was used to judge clinical relevance.

**Clinical interpretation:** To facilitate clinical interpretation of the network findings, two integrated visualizations were developed. First, a Connectivity–Change Matrix was constructed by plotting each of the 17 symptom and functioning nodes on two orthogonal dimensions: baseline network centrality (Bridge Expected Influence at T0, y-axis) and clinical improvement during rehabilitation (standardized effect size, x-axis). The Connectivity–Change Matrix is presented as an exploratory visualization. The effect size for each node was computed as the absolute posterior median difference Δ divided by the standard deviation at T0 (|Δ|/SD T0), providing a scale-free measure comparable across EORTC QLQ-C30 and HADS subscales. The matrix was divided into quadrants using Cohen’s d = 0.5 as the vertical anchor (conventional threshold for a medium effect) and the median Bridge EI across all 17 nodes (0.56) as the horizontal anchor. Second, a Symptom Improvement Ranking was constructed by sorting all 17 nodes by standardized effect size; bar color encodes baseline Bridge EI (darker = more central), allowing simultaneous assessment of magnitude of observed change and network importance. The quadrant boundaries are heuristic and do not represent clinically validated cut-offs. The matrix is intended to illustrate which domains combine higher network connectivity with larger observed change, not to establish validated treatment-priority categories. Both figures were generated in R using ggplot2.

**Sensitivity analyses:** To explore the transdiagnostic generalisability of the pooled symptom-network structure, BMA networks were estimated separately for each diagnostic subgroup with at least 80 complete cases at T0 (ten entities; 15.000 iterations). For each subgroup, structural consistency was quantified as the Pearson correlation between the subgroup and pooled BMA-weighted edge weights across all 136 edges. Because each subgroup also contributes to the pooled network (most strongly for the largest entity), this correlation is not fully independent. To address this, we additionally computed a leave-one-out estimate in which each subgroup’s edge weights were correlated with a pooled network re-estimated after excluding that subgroup [39]. These analyses were post hoc and exploratory.

All analyses were performed using R version 4.5.3 in RStudio version 2026.01.1 Build 403 (“Apple Blossom” Release; Posit Software, PBC) on Windows.

## 3. Results

### 3.1. Sample Characteristics

Of 5571 cancer survivors who met the predefined inclusion criteria (adults completing inpatient rehabilitation; first admission only; T0-to-admission interval ≤ 56 days; no termination within the first three days), 505 (9.1%) were excluded because of incomplete patient-reported outcome data on at least one of the 17 network variables at admission or discharge. The final complete-case analytical sample comprised 5066 cancer survivors (mean age 59.9 years, SD 12.0; 64.0% female). The most common diagnosis was breast cancer (n = 1905; 37.6%), followed by hematological malignancies (n = 516; 10.2%), prostate cancer (n = 445; 10.2%), and female genital cancers (n = 358; 7.1%). For details, see Table 1.

**Table 1:** Patient Characteristics (*n* = 5066).

Characteristic	*n*	%
Age (years)		
≤50	955	18.9%
51–60	1724	34.0%
61–70	1425	28.1%
71–80	730	14.4%
>80	232	4.6%
Mean (SD)	59.9 (12.0) years	
Sex		
Female	3242	64.0%
Male	1824	36.0%
Primary cancer diagnosis		
Breast cancer (C50)	1905	37.6%
Hematological malignancies (C81–96)	516	10.2%
Prostate cancer (C61)	445	10.2%
Uterine and ovarian cancers (C53–56)	358	7.1%
Colon cancer (C18–19)	263	5.2%
Head and neck cancers (C00–14; C30–32)	245	4.8%
Lung cancer (C33–34)	206	4.1%
Rectal cancer (C20–21)	157	3.1%
Gastric cancer (C16)	112	2.2%
Other malignant diagnoses	859	17.0%

To assess potential selection bias, included (*n* = 5066) and excluded (*n* = 505) patients were compared in terms of age, sex, and tumor type. The groups did not differ in sex (36.0% vs. 33.7% male; *p* = 0.32) and differed only slightly in age (59.9 vs. 63.8 years; d = −0.32). The distribution of tumor types differed between groups (*p* < 0.001), with lung and upper-gastrointestinal cancers somewhat over-represented and breast cancer under-represented among excluded patients. This may reflect the higher symptom burden and poorer performance status associated with these entities, which could plausibly reduce the likelihood of complete questionnaire data at both timepoints.

Markov Chain Monte Carlo (MCMC) convergence was satisfactory for all estimated models. Effective sample sizes (ESS) exceeded 1000 for all 136 edge parameters at both T0 (range: 13,223–17,409) and T1 (range: 13,166–17,631), confirming adequate posterior exploration with 15,000 sampling iterations. In practical terms, high ESS values indicate that the MCMC chains produced a large number of effectively independent posterior samples, meaning the estimated network edges are unlikely to be driven by poor chain mixing or autocorrelation.

Prior to network estimation, multicollinearity among the 17 variables was examined. The largest absolute pairwise correlation between any two scales was r = 0.69, and all variance inflation factors (VIF) were ≤3.2 (range 1.1–3.2). These results indicated no problematic collinearity, supporting the stability of the estimated partial-correlation network.

### 3.2. Symptom-Level Change

On the level of mean symptom change during rehabilitation, Bayesian paired t-tests indicated strong evidence for improvement across all 17 variables (all BF_10_ > 10; Table 2); however, the clinical relevance of these changes varied substantially. Following the recommendations by Cocks et al. [37], clinically relevant improvement was observed for emotional functioning, social functioning, global QoL, fatigue, and role functioning (all > 10 points difference), while for pain, appetite loss, insomnia, financial impact, and constipation smaller but potentially relevant change (5–10 points) was observed. Both HADS subscales exceeded their MIDs (anxiety 1.8 vs. 1.3; depression 1.9 vs. 1.4). In contrast, several domains showed changes below the MID despite decisive statistical evidence, most clearly dyspnoea (1.4 points; d = 0.05), but also diarrhea, nausea/vomiting, and cognitive functioning.

At admission, 15.0% of patients scored in the probable-case range for anxiety (HADS-A ≥ 11) and 10.4% for depression (HADS-D ≥ 11); using the broader borderline threshold (≥8), 35.9% and 25.8%, respectively. By discharge, the proportion of probable cases had more than halved, to 6.2% for anxiety and 3.8% for depression (borderline: 18.8% and 11.8%). Both reductions in probable caseness were statistically significant (McNemar χ^2^ = 306.5 and 242.0, respectively; both *p* < 0.001).

To characterize mean change during treatment at the individual level, each patient was classified per domain as improved, stable, or deteriorated relative to the minimal important difference (Table 2). Improvement was most common for fatigue (61.8% improved), emotional functioning (58.1%), role functioning (52.4%), social functioning (52.1%), global QoL (51.7%), and the HADS subscales (anxiety 51.3%; depression 49.7%). Emotional functioning and global QoL showed the most favorable balance, with improvement greatly exceeding deterioration (58.1% vs. 6.1% and 51.7% vs. 6.2%, respectively). For several domains, however, a substantial minority of patients deteriorated despite group-level improvement, including cognitive functioning (22.4% deteriorated), dyspnoea (20.4%), insomnia (18.9%), fatigue (17.8%), and pain (17.8%). This indicated genuinely bidirectional change rather than uniform benefit. On the symptom scales with low baseline burden (e.g., nausea/vomiting, constipation, diarrhea, appetite loss), the majority of patients were classified as stable (68–72%), largely reflecting floor effects: patients with little or no symptom burden at admission could not register a further MID-level improvement. Decisive Bayesian evidence of change (all BF_10_ > 100) was therefore present even for domains where most individual patients did not meet the threshold for clinically meaningful improvement.

### 3.3. Symptom Network Structure at Admission

**Figure 1:**
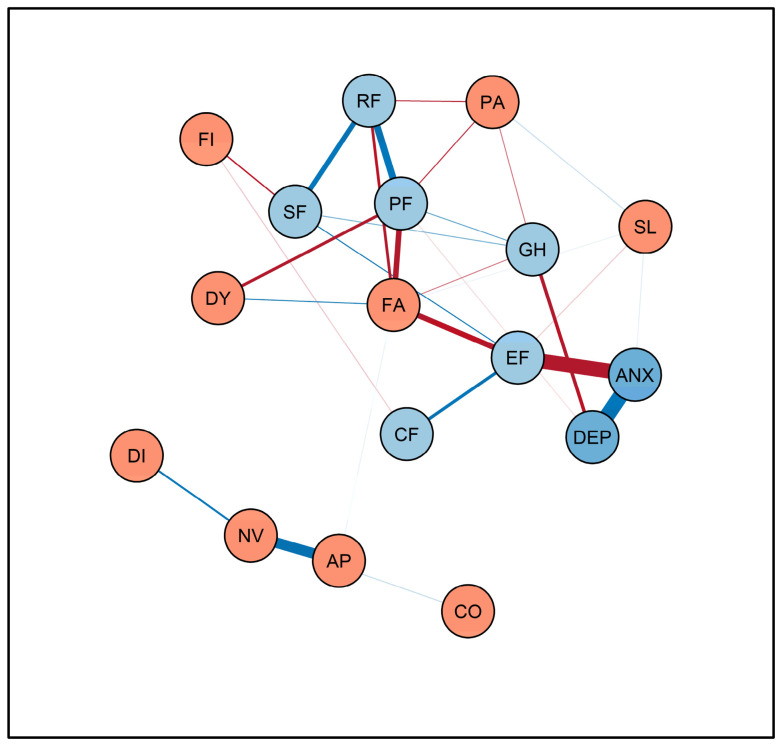
**Bayesian Gaussian Graphical Model Analysis of cancer-related symptoms at admission.** Nodes represent symptoms and functioning domains; edges represent partial correlation coefficients estimated via Bayesian Model Averaging (BMA). Only edges with BMA-weighted partial correlations |r| > 0.10 are shown in the visualization of networks. **Edge Colors**: Blue edges indicate positive associations (e.g., symptom-symptom coupling), while red edges indicate negative associations (e.g., negative (inverse) associations between functioning and symptom domains). Edge thickness and brightness represent the strength of the association. The layout is fixed to the T0 baseline to allow for direct visual comparison of structural changes. Node colors indicate functional domains: light green (functioning), light orange (symptoms), and light blue (psychological distress). **Abbreviations**: **PF**: physical functioning; **RF**: role functioning; **SF**: social functioning; **EF**: emotional functioning; **CF**: cognitive functioning; **GH**: global health status; **FA**: fatigue; **NV**: nausea/vomiting; **PA**: pain; **DY**: dyspnoea; **SL**: insomnia; **AP**: appetite loss; **CO**: constipation; **DI**: diarrhea; **FI**: financial impact; **ANX**: HADS anxiety; **DEP**: HADS depression.

The symptom network estimated at admission (T0) as shown in Figure 1 depicts the characteristic pattern of symptom co-occurrence that defines the lived burden of cancer survivors entering inpatient rehabilitation, i.e., the connections between physical, functional, and psychological impairments that exist before rehabilitation begins. This network structure is therefore relevant far beyond the rehabilitation setting; it maps the symptom interdependencies that oncologists, general practitioners, and psycho-oncologists encounter whenever they assess a cancer survivor’s multidimensional burden.

At admission, 77 of 136 possible edges showed Posterior Inclusion Probabilities (PIPs) ≥ 0.50, and 70 showed PIPs ≥ 0.75, indicating that a large proportion of symptom pairs are meaningfully coupled in this patient population. The five edges with the highest inclusion certainty (all PIPs = 1.000) were between social functioning and financial impact, role functioning and fatigue, emotional functioning and fatigue, nausea/vomiting and appetite loss, as well as anxiety and depression. This pattern is clinically coherent: emotional and physical exhaustion, social life constrained by financial burden, and psychological distress are tightly interwoven upon rehabilitation entry.

The most influential nodes at admission (i.e., those most strongly connected across different symptom clusters) were emotional functioning (bridge EI = 1.103), anxiety (bridge EI = 1.052), and physical functioning (bridge EI = 1.051). These three nodes function as the main “relay stations” in the symptom network: they connect between the somatic, functional, and psychological domains of the patient’s symptom experience. Emotional functioning also showed the largest-magnitude Expected Influence at both timepoints; as detailed below, the sign of this measure depends on scale coding and is therefore not interpreted substantively.

### 3.4. Symptom Network Structure at Discharge

**Figure 2:**
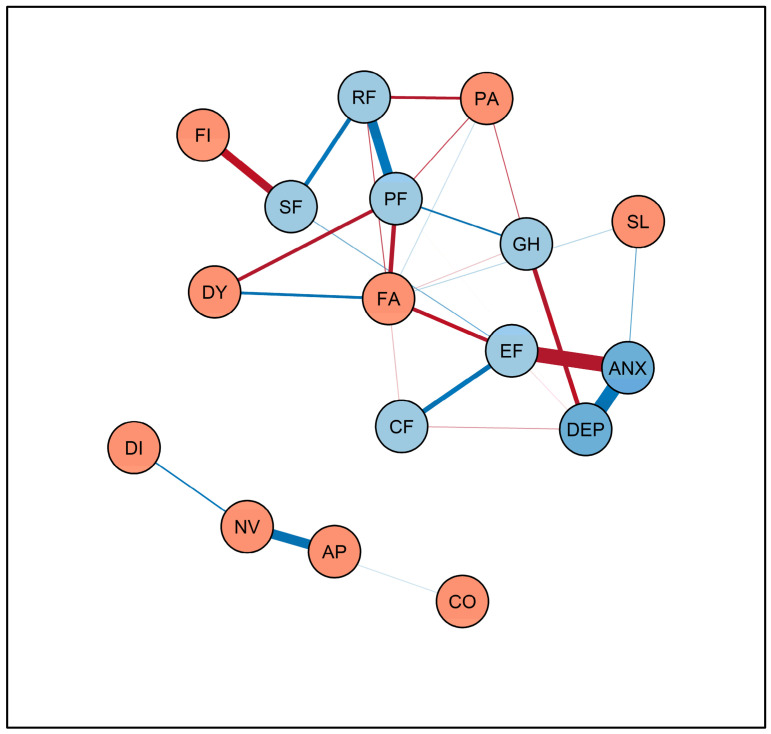
**Bayesian Gaussian Graphical Model Analysis of cancer-related symptoms at discharge (T1)**. Nodes represent symptoms and functioning domains; edges represent partial correlation coefficients estimated via Bayesian Model Averaging (BMA). Only edges with BMA-weighted partial correlations |r| > 0.10 are shown in the visualization of networks. **Edge Colors**: Blue edges indicate positive associations (e.g., symptom-symptom coupling), while red edges indicate negative associations (e.g., negative (inverse) associations between functioning and symptom domains). Edge thickness and brightness represent the strength of the association. The layout is fixed to the T0 baseline to allow for direct visual comparison of structural changes. Node colors indicate functional domains: light green (functioning), light orange (symptoms), and light blue (psychological distress). **Abbreviations**: **PF**: physical functioning; **RF**: role functioning; **SF**: social functioning; **EF**: emotional functioning; **CF**: cognitive functioning; **GH**: global health status; **FA**: fatigue; **NV**: nausea/vomiting; **PA**: pain; **DY**: dyspnoea; **SL**: insomnia; **AP**: appetite loss; **CO**: constipation; **DI**: diarrhea; **FI**: financial impact; **ANX**: HADS anxiety; **DEP**: HADS depression.

The symptom network estimated at discharge (T1) as shown in Figure 2 is qualitatively distinct from the admission network: it is not a general description of cancer survivors, but specifically the symptom architecture of patients who have received 21 days of structured multidisciplinary inpatient rehabilitation. Thus, the discharge network quantifies central changes in the symptom and functioning networks that patients show in oncological rehabilitation.

At discharge, 75 edges showed PIPs ≥ 0.50 and 67 showed PIPs ≥ 0.75. This modest reduction in network density is consistent with a general loosening of symptom coupling alongside the observed mean-level improvements. The results indicate that symptoms did not merely reduce during rehabilitation, but there was also a slight reduction in the degree to which symptoms constrain one another. Critically, the structural position of the three highest-centrality nodes was preserved at T1: emotional functioning maintained the highest bridge EI (1.129, marginally increased), fatigue remained second (0.920), and physical functioning third (0.985).

### 3.5. Rehabilitation-Induced Network Changes

Of the 136 unique edges, 23 (17%) showed 95% HDIs of the posterior difference distribution that excluded zero (for details, see Table 3). When applying a stricter 99% HDI, 14 also excluded zero and 6 reached a posterior probability of direction above 0.999. By contrast, under the magnitude-based ROPE criterion (±0.05), only one edge, namely the social functioning–financial impact coupling, lay entirely outside the region of practical equivalence, while 43 edges showed positive evidence of practical equivalence (95% HDI within ±0.05). Taken together, these analyses indicate that the edge-level changes were reliably directional but generally small: the network underwent limited, mostly minor reorganization against a background of substantial practical stability.

**Table 3:** Edges with Credible Change from Admission (T0) to Discharge (T1).

Edge (Node 1–Node 2)	r (T0)	r (T1)	PIP T0	PIP T1	Δ (T0–T1)	95% HDI	Change at T1
Social functioning–Financial impact	−0.164	−0.276	1.00	1.00	−0.112	[−0.149, −0.076]	**Stronger**
Role functioning–Emotional functioning	−0.059	0.023	1.00	0.16	+0.083	[0.043, 0.120]	**Weaker**
Nausea/vomiting–Anxiety	0.054	−0.022	0.99	0.15	−0.076	[−0.115, −0.038]	**Weaker**
Cognitive functioning–Financial impact	−0.119	−0.054	1.00	0.99	+0.065	[0.027, 0.105]	**Weaker**
Role functioning–Fatigue	−0.196	−0.132	1.00	1.00	+0.064	[0.026, 0.102]	**Weaker**
Cognitive functioning–Depression	−0.061	−0.119	1.00	1.00	−0.058	[−0.098, −0.020]	**Stronger**
Physical functioning–Anxiety	0.076	0.018	1.00	0.10	−0.058	[−0.097, −0.019]	**Weaker**
Fatigue–Anxiety	−0.109	−0.052	1.00	0.98	+0.058	[0.019, 0.096]	**Weaker**
Social functioning–Global QoL	0.133	0.078	1.00	1.00	−0.055	[−0.094, −0.017]	**Weaker**
Pain–Constipation	0.063	0.008	1.00	0.06	−0.055	[−0.094, −0.016]	**Weaker**
Nausea/vomiting–Appetite loss	0.350	0.297	1.00	1.00	−0.054	[−0.088, −0.019]	**Weaker**
Appetite loss–Diarrhea	0.084	0.031	1.00	0.36	−0.054	[−0.092, −0.015]	**Weaker**
Social functioning–Depression	−0.093	−0.040	1.00	0.77	+0.053	[0.013, 0.091]	**Weaker**
Anxiety–Depression	0.443	0.391	1.00	1.00	−0.052	[−0.084, −0.019]	**Weaker**
Emotional functioning–Fatigue	−0.277	−0.230	1.00	1.00	+0.048	[0.012, 0.084]	**Weaker**
Global QoL–Financial impact	−0.022	0.024	0.14	0.18	+0.046	[0.007, 0.084]	**Stronger**
Emotional functioning–Appetite loss	−0.010	−0.056	0.06	0.99	−0.046	[−0.084, −0.007]	**Stronger**
Fatigue–Nausea/vomiting	0.089	0.044	1.00	0.87	−0.046	[−0.084, −0.007]	**Weaker**
Role functioning–Social functioning	0.246	0.204	1.00	1.00	−0.042	[−0.079, −0.005]	**Weaker**
Physical functioning–Financial impact	−0.040	−0.080	0.76	1.00	−0.040	[−0.080, −0.002]	**Stronger**
Role functioning–Appetite loss	0.006	−0.034	0.05	0.49	−0.040	[−0.080, −0.003]	**Stronger**
Emotional functioning–Nausea/vomiting	−0.00	−0.04	0.05	0.71	−0.038	[−0.078, −0.000]	**Stronger**
Emotional functioning–Anxiety	−0.432	−0.397	1.00	1.00	+0.035	[0.000, 0.065]	**Weaker**

Note. Δ = posterior mean difference in BMA-weighted partial correlation (T0 − T1). The sign of Δ indicates the direction of the numerical difference only; because edges may be positive or negative, the sign does not by itself indicate strengthening or weakening. The “Change at T1” column therefore reflects the change in absolute edge weight (|r at T1| − |r at T0|): “Stronger” = increased association strength, “Weaker” = decreased. Edges ordered by |Δ|. The remaining 113 of 136 edges showed no credible change (95% HDI included zero). HDI = Highest Density Interval; PIP = posterior inclusion probability.

Only a small number of edges showed reliable change and are described here as exploratory, hypothesis-generating observations. Only the strengthening of the social functioning–financial impact coupling, which became more strongly negative at discharge (Δ = −0.112), was robust to the conservative practical-equivalence criterion. By discharge, this link had substantially intensified, even though financial difficulties themselves improved only modestly (d = 0.21). In other words, although patients reported somewhat less financial burden after rehabilitation, the burden that remained was more tightly linked to constrained social functioning.

Two further changes were directionally reliable (99% HDI excluding zero) but smaller in magnitude, and are noted as tentative. For one, role functioning became less tightly coupled to both fatigue (Δ = 0.064) and emotional functioning (Δ = 0.082) at discharge. In other words, at admission, a patient’s ability to carry out daily work and activities was strongly dependent on their level of fatigue and emotional distress. By discharge, these dependencies had weakened, indicating patients could maintain better role functioning more independently of their remaining fatigue and emotional burden.

Additionally, the association of nausea/vomiting and anxiety weakened markedly at discharge and effectively dropped out of the network (PIP: 0.988 → 0.148, Δ = −0.076).

The full table of all 136 unique edges is shown in Supplementary Materials Table S1.

**Figure 3:**
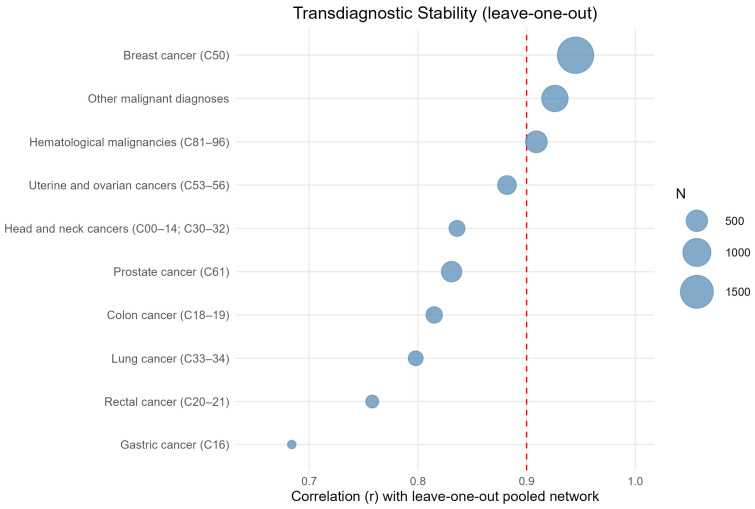
Transdiagnostic structural consistency: leave-one-out correlations between each diagnostic subgroup’s network and the pooled network across all 136 edges.

**Transdiagnostic consistency:** Across the ten diagnostic subgroups (each n ≥ 80), the symptom-network structure was highly consistent with the pooled network. Leave-one-out correlations (which means that each subgroup’s edge weights were estimated against the pooled network without that subgroup) ranged from r = 0.68 (gastric, *n* = 112) to r = 0.95 (breast, *n* = 1905), with 7 of 10 subgroups exceeding r = 0.80 and 9 of 10 exceeding r = 0.75 (Figure 3). The correction for each subgroup’s own contribution to the pooled estimate was small (≤0.03), indicating that the high cross-diagnostic consistency was not an artifact of overlap. These analyses are exploratory: because subgroups differ systematically in unmeasured clinical characteristics (e.g., stage, treatment), the observed consistency is structural and descriptive rather than evidence of a confounding-adjusted shared architecture.

### 3.6. Centrality

Table 4 presents Expected Influence (EI) and bridge EI for all nodes at T0 and T1. EI measures whether a node tends to activate or suppress its neighbors (negative EI = inverse-association pattern), while Bridge EI measures how strongly a node connects different symptom clusters. Emotional functioning showed the largest-magnitude Expected Influence at both timepoints. Because the network combines functioning scales (higher = better) and symptom scales (higher = worse), the sign of Expected Influence is dependent on scale coding; a sensitivity analysis recoding all scales in a common direction left the ranking of nodes by Bridge Expected Influence unchanged (Spearman ρ = 1.00) but reversed the sign of Expected Influence, confirming that the sign—and any ‘buffering’ interpretation based on it—reflects coding direction rather than a substantive mechanism. We therefore base interpretation on Bridge Expected Influence (coding-invariant) and on the magnitude of centrality rather than on the sign of Expected Influence. Bridge EI was highest for emotional functioning (T0: 1.103; T1: 1.129), anxiety (T0: 1.052; T1: 0.951), and physical functioning (T0: 1.051; T1: 0.985). Thus, these three nodes are identified as the primary connectors between the functioning, symptom, and psychological communities of the network. No node showed a qualitative change in its centrality pattern across the two timepoints, reinforcing the interpretation of overall structural stability.

In a sensitivity analysis recoding all scales to a common direction (higher = greater burden), the rank ordering of nodes by Bridge Expected Influence was identical to the original analysis (Spearman ρ = 1.00): emotional functioning, anxiety, physical functioning, and fatigue remained the most central bridging nodes. The magnitude-based ranking of Expected Influence was moderately preserved (ρ = 0.56), but the sign of Expected Influence changed under common coding, confirming that the sign is dependent on scale direction while the centrality ranking remains unchanged. Substantive interpretation therefore focuses on Bridge Expected Influence and on the magnitude of centrality rather than on the sign of Expected Influence.

Reassigning financial difficulties to the functioning/social community left the Bridge Expected Influence ranking essentially unchanged (Spearman ρ = 0.96), with the most central nodes, i.e., emotional functioning, anxiety, physical functioning, and fatigue, maintained their positions. Only the bridge centrality of financial difficulties itself changed, as expected. Re-estimating the network without financial difficulties likewise preserved the centrality ranking of the remaining nodes (ρ = 0.90). The network’s centrality structure was therefore robust to the classification of financial difficulties.

Centrality estimates were accompanied by 95% credible intervals derived from the posterior (Table 4). At admission, the four most central bridging nodes—emotional functioning, fatigue, anxiety, and physical functioning—had credible intervals clearly separated from those of the lower-centrality nodes, though overlapping with one another, so these four are reliably identified as the most central without a reliable rank order among them. Emotional functioning retained the highest bridge centrality at discharge, with overlapping intervals across timepoints indicating stable centrality. Fatigue and anxiety showed modest reductions in bridge centrality at discharge.

### 3.7. Clinical Interpretation

**Figure 4:**
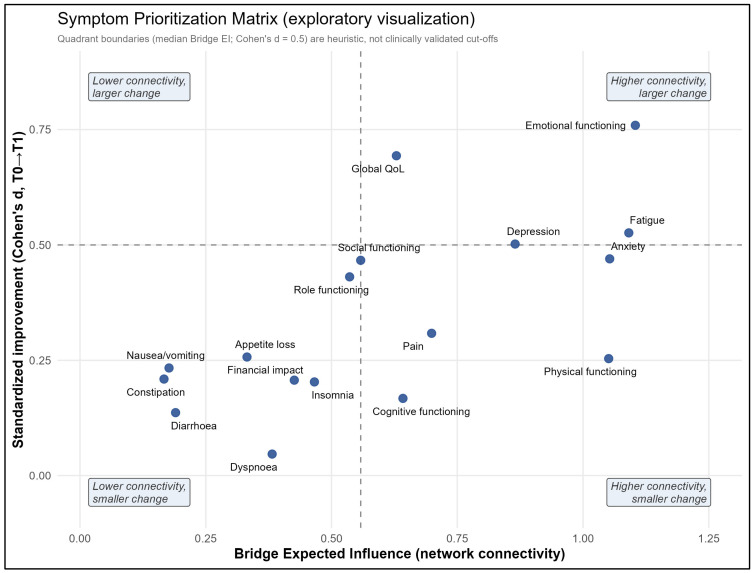
Connectivity–Change Matrix: Baseline network centrality vs. clinical improvement during rehabilitation. Each circle represents one of the 17 symptom and functioning domains assessed at admission (T0) and discharge (T1). In this figure, the bridge expected influence, i.e., a network metric that captures how strongly a given symptom connects different symptom clusters, is plotted against the standardized effect size, which shows how much a symptom improved during the rehabilitation. The vertical line represents Cohen’s d = 0.50, the conventional threshold for a clinically meaningful medium effect. The horizontal line represents the median network centrality across all 17 domains.

To facilitate the clinical interpretation of the network outcomes, a Connectivity–Change Matrix was designed. In Figure 4, the baseline network centrality (bridge EI) is combined with the observed magnitude of symptom and functioning change during rehabilitation. This figure is presented as an exploratory visualization combining two descriptive dimensions—each domain’s Bridge Expected Influence (network connectivity) and its standardized improvement during rehabilitation. On the horizontal axis, it displays how much each domain improved during the 21-day inpatient rehabilitation program, expressed as a standardized effect size. The vertical axis shows how central each domain was within the symptom network at admission, measured by bridge EI, i.e., a network metric that captures how strongly a given symptom connects different symptom clusters (e.g., physical symptoms and psychological distress). Domains in the upper-right quadrant (combination of higher connectivity with larger observed change) were both highly influential within the network at baseline and showed large clinical improvement during rehabilitation. Domains in the upper-left quadrant (high connectivity with smaller observed change) were highly central in the network but showed comparatively limited improvement, suggesting that despite their structural importance these symptoms may require more intensive or specifically targeted intervention beyond the current standard program.

The two variables that were identified as a combination of higher connectivity with larger observed change were emotional functioning (effect size d = 0.83; bridge EI = 1.103) and fatigue (d = 0.56; bridge EI = 1.091), i.e., combining high structural connectivity in the symptom network and largest responses to treatment. In contrast, anxiety and physical functioning were identified as highly connected with smaller observed change since they both showed high network centrality at baseline (bridge EI = 1.052 and 1.051, respectively) but only rather low improvement during rehabilitation (d = 0.28 and 0.25, respectively). Global health and social functioning showed medium-to-large improvement with moderate centrality, while gastrointestinal symptoms, dyspnoea, and constipation were clustered in the lower-left quadrant, showing both low centrality and small standardized change.

**Figure 5:**
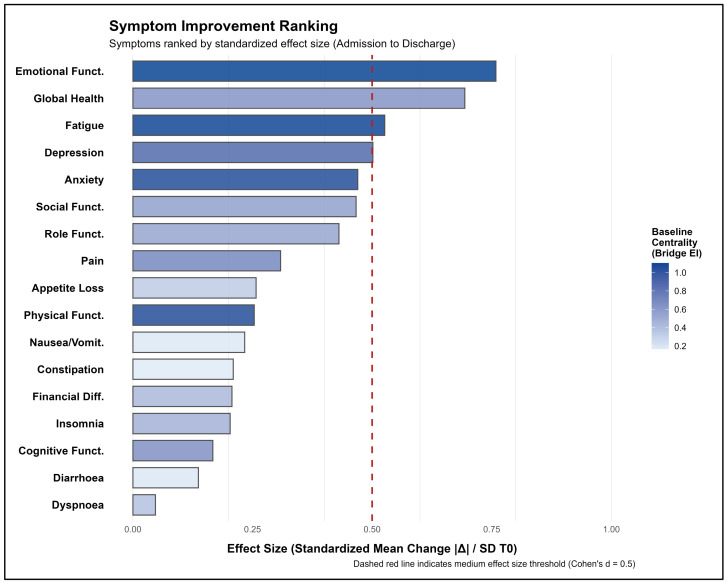
Symptom Improvement Ranking: Standardized clinical change from admission to discharge. All 17 symptom and functioning domains are ranked by the magnitude of improvement achieved during the 21-day inpatient rehabilitation program, expressed as a standardized effect size (absolute mean change divided by the standard deviation at admission). The dashed red line marks Cohen’s d = 0.50, a conventional threshold for a clinically meaningful medium effect. Bar color indicates each domain’s baseline network centrality (bridge expected influence at admission): darker blue bars represent domains that were more central—more connected across different symptom clusters—within the symptom network at the time of admission.

To further identify variables with high potential to change, a symptom improvement ranking was plotted in Figure 5. All 17 variables were listed based on the observed effect size of symptom improvement, with bar color indicating baseline centrality. This color-coding system enables not only to appreciate the extent to which each symptom has improved, but also to assess its structural significance within the overall symptom burden. Largest changes during treatment were observed for emotional functioning, global health, and fatigue (all d > 0.50). While depression (d = 0.27), anxiety (d = 0.28), and physical functioning (d = 0.25) showed small-to-medium effects, the darker bar color highlights their strong connectivity in the baseline network.

For example, the domains of physical functioning and anxiety rank in the middle of the list in terms of improvement, yet their dark bar color signals that both were among the domains with the highest network centrality at admission. This suggests that targeted interventions to treat these symptoms are likely to make a disproportionately large contribution to overall symptom relief. Emotional functioning, global health, and fatigue were the three domains with the largest standardized improvements, all exceeding the medium effect threshold. In contrast, dyspnoea showed the smallest improvement, consistent with a more peripheral role in the symptom network.

## 4. Discussion

The aim of this study was to examine the interdependence between health-related symptoms and functional abilities that shape the lives of cancer survivors, as well as to assess systematic changes in the network structure during an inpatient cancer rehabilitation program. A very large and diverse sample of cancer survivors who participated in a 21-day, multidisciplinary inpatient cancer rehabilitation program was instrumental to this effort. To our best knowledge, this is the largest Bayesian Gaussian Graphical Model in this clinical context of cancer survivorship so far [24,40].

Three main findings emerged. First, the data indicated that the inpatient rehabilitation treatment was associated with clinically meaningful improvements across all 17 assessed symptom and functioning domains. The largest effects were found for emotional functioning, global health status, and fatigue. Second, the underlying architecture between the symptom and functioning scales was largely preserved throughout the treatment. Of all 136 edges in the network, 82% showed no credible change, which indicated a robust and stable network topology as a result of the patients’ experiences related to their cancer and the respective treatments, which may include surgery, radiation therapy, chemotherapy, immunotherapy, or combinations thereof. Third, emotional functioning and fatigue were identified as domains combining high connectivity with larger observed change, while anxiety and physical functioning were identified as candidate domains for future intervention studies based on their central role in the networks.

Overall, the observed pattern of improvement in symptoms and functional capabilities was closely aligned with our previous analyses [20,21,41] which were partially based on the patient collective included in this study. In these studies, we investigated the extent to which rehabilitation can improve individual symptoms and functions in patients with various underlying tumor entities or in different age groups. However, the present study, which was designed differently, adds three important dimensions.

For one, the sample size was substantially increased as compared to the last analysis, with almost 700 additional patients due to the extended observation period. Due to the large sample size, the posterior distributions were exceptionally precise, and even the smallest observed improvements were estimated with complete directional certainty (posterior probability of direction > 99.9% for all 17 domains). Second, the use of standardized effect sizes across the EORTC QLQ-C30 and HADS subscales in this study allowed us to directly compare the magnitude of change across instruments. In our present analysis, emotional functioning and the global health status showed the largest effects, followed by fatigue, social functioning, and role functioning. Third, both depression and anxiety exceeded the established minimal important differences, and their effect sizes are now directly comparable with somatic scales, which has not been feasible in the previously applied analysis in our patient samples.

In our previous analysis [21], we noted a consistent pattern of improvement across different tumor entities. Furthermore, since we could show in another analysis [25] that the applied ePRO system in this center [26] leads to high-quality and representative data across age groups, we can place additional confidence in the robustness of the presented results. In light of our previous analyses, we assume that the present study adds to the coherent evidence base for multimodal inpatient cancer rehabilitation. In addition to the previously documented robust mean-level improvements of symptoms and functioning, we now present data on the structural and network-level based evaluation of treatment outcome.

Compared to improvements in HRQOL functioning and somatic symptom domains, reductions in anxiety and depression were less pronounced. This finding is consistent with previous rehabilitation studies in cancer survivors reporting smaller improvements in psychological distress than in physical functioning and symptom burden [21]. In particular, fear of cancer recurrence has repeatedly been identified as one of the most prevalent and enduring unmet needs among cancer survivors and is closely linked to anxiety, uncertainty, and reduced quality of life [42]. Similarly, previous work has emphasized that ongoing psychosocial concerns—including altered body image, uncertainty about the future, occupational disruption, and financial strain—may continue to shape emotional well-being long after treatment completion [43,44,45]. These broader survivorship-related stressors may be less amenable to change during a relatively brief inpatient rehabilitation period and could explain why anxiety remained among the most central symptoms within the network despite overall clinical improvement.

The network-based perspective we take in this paper helps to further refine this observation. Our results indicate that the comparatively modest change in psychological distress does not actually reflect structural resistance, but rather they show that anxiety and depression occupy high-centrality positions within the symptom and functioning networks, where change is mediated through many interconnected pathways rather than concentrated in one direction. This suggests that even comparatively small improvements in anxiety and depression may still be clinically important, as these symptoms were strongly connected to, and thus influence or modify multiple other symptom and functioning domains within the network. From a network perspective, interventions targeting highly central symptoms may therefore have broader effects on the overall symptom burden than would be expected from mean-level symptom change alone. Network theory would expect that changes in high-centrality nodes have broad propagating effects across connected symptoms [16]. To prove this assumption, prospective network studies with repeated assessments are needed to formally test whether anxiety and depression reductions during rehabilitation predict cascading improvements in somatic domains.

The main structural finding of this study was the high stability of the symptom network throughout the rehabilitation period, with 82% of all edges showing no credible change over time. It is important to highlight that this stability does not reflect an absence of clinical change or improvement, respectively, but rather indicates that the connections and patterns between symptoms and psychosocial functions of cancer survivors appear to be a robust feature of cancer survivorship surveys, which is largely independent of the absolute level of the observable symptom burden. In our study, the multimodal inpatient rehabilitation program was in fact associated with substantial improvement in both symptoms and functions and thus shifted the state of the system substantially without, however, fundamentally reorganizing its architecture. These results are in line with the conceptual distinction between symptom level and symptom structure in network psychopathology [13] which includes the assumption that symptom topology can remain stable while mean symptom levels change substantially.

Several alternative explanations for the observed structural stability should be considered. First, the absence of detected change in the majority of edges is not equivalent to positive evidence of equivalence; with 136 simultaneous edge comparisons, some genuine changes may have gone undetected, and ‘no credible change’ should be read as the absence of strong evidence for change rather than proof of its absence. Second, the rehabilitation treatment of 21 days may be too short for the architecture of symptom interrelationships to reorganize, even where mean symptom levels improve substantially. Structural change may unfold over longer survivorship trajectories that a single inpatient episode cannot capture. Third, methodological features of the analysis favor apparent stability: the node layout was fixed to the admission network to permit visual comparison, which by design makes the two networks look similar, and the very large sample yields highly precise posterior estimates that are individually stable across timepoints. Fourth, because the networks represent between-person conditional associations at two separate timepoints rather than within-person dynamics, they may be relatively insensitive to individual-level reorganization that does not alter the group-level association structure. Taken together, these considerations suggest that the observed stability is best interpreted as evidence that the *group-level* symptom architecture is robust over a short rehabilitation episode, rather than as evidence that symptom networks are immutable. However, longitudinal designs with multiple assessments and within-person modeling will be needed to determine whether and over what timescale these structures change.

Against this background of overall structural stability, a small number of edges showed reliable change and are described here as exploratory, hypothesis-generating observations (of the 23 credibly changed edges, only one was robust to the conservative practical-equivalence criterion). First, role functioning became less tightly coupled to both fatigue and emotional functioning at discharge, suggesting that patients’ capacity to carry out daily activities became somewhat more independent of their remaining fatigue and emotional burden as physical capacity improved—consistent with the intended effects of the exercise, fatigue-management, and occupational-therapy components of rehabilitation. This corresponds to a finding in our previous study, in which the median fatigue score decreased by more than 16 points on the EORTC QLQ-C30 scale during rehabilitation [21].

Second, the largest credible network change—and the only one robust to the conservative practical-equivalence (ROPE) criterion—was a strengthening of the association between social functioning and financial difficulties, which became more strongly negative at discharge (r = −0.164 → −0.276; Δ = −0.112). At admission, reduced social participation was already associated with greater financial burden after controlling for all other domains; by discharge, this inverse coupling had intensified, even though the absolute level of financial difficulties improved only modestly. In other words, although patients reported somewhat less financial burden after rehabilitation, the burden that remained was more tightly linked to constrained social functioning. This may reflect a heightened salience of financial concerns as patients begin to anticipate the return to everyday life, or a tightening of the psychosocial link between financial strain and social participation that the residential setting does not resolve. Previous survivorship research has shown that financial toxicity is closely associated with anxiety, depression, and reduced quality of life [46,47], and that subjective financial strain is strongly shaped by emotional processing rather than objective circumstances alone [48]. Whether this strengthened coupling persists, intensifies, or attenuates after discharge is an important question for follow-up research.

Because the absolute level of financial difficulties improved only modestly, this strengthened coupling is unlikely to reflect resolution of patients’ objective financial situation; rather, it may indicate that as patients prepare to leave the supportive residential setting, residual financial concerns become more tightly bound to anticipated constraints on social participation. Whether this intensified coupling persists, attenuates, or reverses after patients return to their everyday environments is an important question for follow-up research, since a strengthening that endured would identify financial–social strain as a target for post-discharge support. Third, the nausea/vomiting–anxiety association weakened markedly at discharge and effectively dropped out of the network. At admission, residual nausea was closely tied to anxiety, which is consistent with conditioned or anticipatory nausea, a well-documented phenomenon in patients with prior chemotherapy [49]. In addition, gastrointestinal symptoms including nausea may be associated with digestive disorders such as dumping syndrome and malnutrition in patients who have undergone gastric surgery [50]. They can also be due to conditions causing intestinal obstruction, such as peritoneal carcinomatosis, mesenteric adhesions or autonomous peripheral neuropathy [51,52,53]. The loosening of this coupling over rehabilitation suggests that, as gastrointestinal symptoms were managed, their anxiety-linked component diminished, which may indicate that the program’s symptom-control and psycho-oncological components were effective in attenuating this association. Previous studies have shown that behavioral and psycho-oncological interventions may help reduce anticipatory nausea and associated distress [49,54].

A recurring question when interpreting network figures concerns edges that appear to be missing despite well-established clinical associations. In our sample, this could, for example, be observed for the association between fatigue and depression, or dyspnoea and anxiety. While both clinical experience and empirical evidence would suggest an association between these variables, no direct connection between the variables can be found in our networks. It is important to highlight that the absence of these direct links is not an analytical artifact. Indeed, they are very meaningful findings that allow us to illustrate one of the core strengths of Gaussian Graphical Models over conventional correlation-based analyses. In the networks displayed in this study, every edge represents a partial correlation, i.e., the unique pairwise association after statistically controlling for all other nodes simultaneously. Therefore, once we include the shared variance, which is explained by emotional functioning, physical functioning, pain, and insomnia, the direct association between fatigue and depression does not add any substantial unique information to the system. This does not indicate that patients with fatigue are not depressed since the bivariate correlation of these scales is substantial. It does however show that the association between fatigue and depression is fully accounted for by their shared connections to other nodes in the network (most prominently emotional functioning, but also physical functioning, insomnia, and pain), thus leaving no unique residual association between them. This interpretation is consistent with previous psycho-oncological research suggesting that cancer-related fatigue and depressive symptoms substantially overlap, while at the same time representing partially distinct constructs with different biological, psychological, and functional correlates [5,55]. Both symptom domains share important affective and behavioral features, yet may arise from partly different underlying mechanisms and therefore show different patterns of association within the broader survivorship symptom network [56].

Conceptually, the emotional functioning scale used in the EORTC QLQ-C30 differs from the HADS depression subscale. While the emotional functioning scale primarily captures broader aspects of emotional distress and affective dysregulation, the HADS depression subscale was specifically developed to assess anhedonic depressive symptomatology while minimizing contamination by somatic symptoms such as fatigue, sleep disturbance, or appetite changes commonly observed in physically ill populations [57]. Accordingly, the HADS depression scale focuses predominantly on reduced positive affect, loss of interest, and diminished enjoyment, whereas emotional distress related to cancer-related symptom burden may be more strongly reflected in the broader emotional functioning construct of the EORTC QLQ-C30. What the network findings suggest can be interpreted as a clinically plausible sequential pathway: fatigue and depressive symptomatology are connected only indirectly through emotional functioning. This finding is consistent with emotional functioning’s role as the primary bridge node between somatic and psychological symptom clusters, and supports prioritizing emotional functioning interventions as the most structurally efficient route to reducing both fatigue-related distress and clinical depression in this population. However, the cross-sectional design of this study does not permit inference about direction.

The same reasoning can be applied to the absence of the direct connection between dyspnoea and anxiety. In our network, it could be observed that after controlling for physical functioning, fatigue, and insomnia, dyspnoea was not directly associated with anxiety. It should be noted that the term “dyspnea” does not generally refer to dyspnea at rest. During rehabilitation, many patients report exercise-induced dyspnea resulting from a lack of physical activity after sometimes long and exhausting periods of cancer treatment, which is expected to be improved through medical exercise therapy with endurance training [58]. Hence, dyspnoea does not always lead to anxiety or fear. The link between the two variables is indirect and might be explained by a clinically plausible sequential pathway: dyspnoea, physical functioning and anxiety are mutually associated, such that the dyspnoea–anxiety association is statistically accounted for by their shared connection with physical functioning. Whether this reflects a mediated pathway cannot be determined from cross-sectional data and remains a hypothesis for prospective testing; if such a pathway exists, respiratory physiotherapy might be associated with psychological benefit through this indirect route. It is well established that physical activity may contribute to the reduction in psychological distress in cancer patients [59,60].

Both of those network-based findings have direct clinical implications. Based on these results, we assume that interventions targeting emotional functioning and physical functioning may simultaneously improve the co-occurrence of fatigue with depression as well as dyspnea with anxiety in a more effective way than approaches that target those symptom pairs directly. These observations are in accord with the general concept of oncological rehabilitation, which is generally performed in a multimodal fashion, combining physical and psychosocial treatment modalities [18,19]. Identifying the underlying network of symptoms and mechanisms can therefore be helpful in optimizing effective combinations of treatment methods.

We aimed to identify variables that are particularly important in inpatient cancer rehabilitation. To achieve these goals, we accounted for both the centrality of the variables in the networks of symptoms and functions as well as the strength of mean symptom change that was observed during the treatment. Based on these assumptions, one key variable was emotional functioning, which showed the highest bridge centrality to other domains at both timepoints, thus identifying it as the structurally most prominent node in the network. Its high bridge centrality identifies it as the structurally most prominent connector between somatic and psychological domains, consistent with the broader literature on emotional resources in cancer survivorship [61]. In combination with the large clinical improvement observed for emotional functioning, it was identified as both a “strategic success” of the treatment as well as a potential key target to facilitate effective inpatient cancer rehabilitation.

In contrast, anxiety and physical functioning were identified as clinically important counterpoints. Both variables showed very high bridge centrality, which indicated they were primary connectors between the functioning and symptom domains as well as the psychological distress domain within the network. However, for both variables, below-medium treatment effect sizes were observed. Based on these observations, both variables combined high bridge centrality with comparatively small observed change, making them candidate domains for further investigation as intervention targets in future studies. Because of anxiety’s central position, it is plausible—though not demonstrable from these cross-sectional data—that improvement in anxiety could be associated with broader network benefit. Even so, this remains a hypothesis requiring prospective testing. However, the results of this study indicate that, within the current multimodal treatment program, there is room for improvement regarding the reduction in the patients’ anxiety. Our findings could be understood as an argument for the systematic integration of anxiety-focused psychological interventions as a structural component of the inpatient rehabilitation program. In contrast, the limited effect sizes of physical functioning despite its high network centrality may reflect ceiling effects in this domain rather than insufficient program intensity. This warrants specific attention since the different cause for the mismatch between network centrality and observed effect sizes during treatment in both cases also leads to substantially different clinical implications.

### Limitations

Several limitations of this study should be acknowledged. For one, the study is based on a single-center observational dataset from one Austrian rehabilitation facility with a specific multidisciplinary program structure. The observed symptom-network architecture and its stability may not generalize to centers with different patient populations, treatment intensities, or program designs, and replication across multiple centers is needed. Second, the design was observational and uncontrolled, without a control group. Therefore, improvements should be viewed primarily as an association and need not be causally attributed to rehabilitation. Natural recovery over time cannot be excluded. Third, the analysis used data from two timepoints only. Our models represent between-person conditional associations which are estimated separately at admission and at discharge. This means that they do not capture within-person temporal dynamics (i.e., intra-individual change in networks over time). In other words, since between-person association networks of the whole patient group were estimated separately at admission and discharge, they do not show how symptoms influence each other within any individual patient over time. Thus, statements about how improvement in one symptom may spread to others are theoretical predictions of network theory and cannot be tested with the present cross-sectional, two-timepoint design. Fourth, no post-discharge follow-up assessments were included, so it remains unknown whether the symptom improvements and the observed structural changes, e.g., the decoupling of social functioning from financial difficulties, persist, attenuate, or reverse once patients return to their everyday environments. The observed patterns have to be verified with longitudinal study designs. Fifth, the analysis was restricted to complete cases to facilitate comparability of networks. Excluded patients were on average slightly older and more often had lung or upper-gastrointestinal cancers, so the sample may modestly under-represent the most severely burdened survivors. Sixth, several clinical variables that strongly influence symptom burden in cancer survivors, including cancer stage, time since diagnosis, recurrence and metastatic status, and treatment modality (surgery, systemic therapy, radiotherapy, endocrine therapy), were not captured in the underlying routine-care dataset and were therefore unavailable for analysis. Consequently, the networks could not be adjusted for disease- and treatment-phase heterogeneity. This is an important limitation: the transdiagnostic structural consistency we report should be interpreted as descriptive rather than as evidence of a confounding-adjusted, mechanism-level shared architecture, since diagnostic subgroups differ systematically in these clinical characteristics. Future studies linking patient-reported outcomes to clinical and treatment data are needed to determine whether the observed network structure persists after accounting for disease stage and treatment phase. Seventh, the nodes in our models are aggregated scale scores rather than individual symptoms, so their network centrality may partly reflect measurement structure and conceptual overlap (for example, between the EORTC emotional functioning scale and the HADS subscales) rather than mechanistic influence. Centrality findings should therefore be regarded as hypothesis-generating. Eighth, the baseline and discharge assessments differed in setting and device: the baseline was completed at home via a web portal, whereas the discharge assessment was completed on-site on a tablet. Differential assessment context, which may include the impact of social desirability, the presence of staff, or positive expectations toward the program at discharge, could in principle have influenced reported scores. Such effects would be expected to act primarily on absolute symptom levels rather than on the conditional-association structure that is the focus of the present network analysis, but a contribution to the observed mean-level changes cannot be ruled out. Finally, standardized effect sizes were computed using the baseline standard deviations of the present analytical sample rather than an external reference distribution. The resulting magnitudes are therefore sample-dependent and should be compared across instruments with this in mind. Reassuringly, the sample’s baseline functioning and symptom levels were consistent with the expected pattern of impairment relative to published EORTC general-population norms [62], indicating that the analytical sample is representative of an inpatient cancer-rehabilitation population, supporting the interpretive validity of the magnitude estimates.

## 5. Conclusions

During rehabilitation, patients showed clinically meaningful improvements across all domains of symptom burden and functioning, against a background of largely stable symptom network architecture. Emotional functioning and anxiety emerge as structurally central nodes at both admission and discharge, identifying them as candidate domains warranting further investigation in intervention studies. Exploratory subgroup analyses provide preliminary evidence of a broadly shared network structure across cancer entities, which requires confirmation in independent samples. These findings contribute a novel network-level perspective on oncological rehabilitation and provide a framework for future targeted intervention research.

## Figures and Tables

**Table 2:** Mean symptom and functioning changes during inpatient cancer rehabilitation (N = 5066).

Scale	Mean T0	(SD)	Mean T1	Change	% Change	d	95% HDI	BF_10_	% Improved	% Stable	% Deteriorated
Emotional functioning	60.7	(24.0)	78.9	18.2	30.1%	0.76	[17.6, 18.8]	>100	58.1	35.9	6.1
Global QoL	60.8	(19.1)	74.0	13.2	21.7%	0.69	[12.7, 13.7]	>100	51.7	42.2	6.2
Social functioning	62.0	(29.5)	75.7	13.8	22.2%	0.47	[13.0, 14.5]	>100	52.1	30.9	17.0
Role functioning	61.2	(28.8)	73.6	12.4	20.3%	0.43	[11.7, 13.2]	>100	52.4	30.4	17.3
Physical functioning	76.5	(20.1)	81.6	5.1	6.7%	0.25	[4.7, 5.5]	>100	30.7	59.8	9.5
Cognitive functioning	74.0	(25.5)	78.2	4.3	5.8%	0.17	[3.7, 4.9]	>100	36.2	41.4	22.4
Fatigue	48.3	(25.5)	34.9	13.5	27.9%	0.53	[12.8, 14.1]	>100	61.8	20.4	17.8
Pain	37.1	(27.9)	28.4	8.6	23.3%	0.31	[8.0, 9.3]	>100	44.8	37.3	17.8
Appetite loss	17.6	(28.3)	10.3	7.3	41.4%	0.26	[6.6, 8.0]	>100	23.8	67.9	8.3
Nausea/vomiting	8.5	(16.9)	4.5	4.0	46.6%	0.23	[3.5, 4.4]	>100	22.0	70.8	7.3
Constipation	15.5	(26.7)	9.9	5.6	36.2%	0.21	[4.9, 6.2]	>100	20.5	71.5	8.1
Financial difficulties	24.9	(31.7)	18.3	6.6	26.4%	0.21	[5.9, 7.3]	>100	24.9	64.0	11.1
Insomnia	44.7	(33.3)	37.9	6.8	15.2%	0.20	[5.9, 7.6]	>100	33.7	47.5	18.9
diarrhea	13.6	(25.0)	10.2	3.4	25.1%	0.14	[2.8, 4.1]	>100	17.9	71.8	10.3
Dyspnoea	28.9	(30.4)	27.5	1.4	4.9%	0.05	[0.7, 2.2]	15.1	23.4	56.2	20.4
Depression (HADS)	5.3	(3.8)	3.4	1.9	35.6%	0.50	[1.8, 2.0]	>100	49.7	41.5	8.7
Anxiety (HADS)	6.4	(3.8)	4.6	1.8	28.0%	0.47	[1.7, 1.9]	>100	51.3	36.3	12.4

Note. EORTC QLQ-C30 scales range 0–100; HADS subscales range 0–21. For functioning scales and global health status, higher scores indicate better functioning; for symptom scales and HADS subscales, higher scores indicate greater burden. Mean T0 = score at admission; Mean T1 = score at discharge. SD = standard deviation; Change = absolute improvement expressed as a positive value throughout (T1−T0 for functioning scales; T0−T1 for symptom scale and HADS). % Change = absolute change as percentage of the T0 mean. d = standardized effect size (|posterior median Δ|/SD T0), comparable across EORTC scale and HADS. 95% HDI = 95% Bayesian Highest Density Interval of the posterior median difference. BF_10_ = Bayes factor in favor of change; “>100” denotes decisive evidence, with exact values omitted as their magnitude is dominated by the large sample size. All Bayesian paired *t*-tests used the default Cauchy prior (scale = √2/2; BayesFactor v0.9.12.4.8). Posterior probability of direction >99.9% for all 17 domains. Domains ordered by effect size within the EORTC QLQ-C30 functioning and symptom domains and the HADS (descending). % Improved/Stable/Deteriorated = proportion of patients whose individual change met the minimal important difference (MID) for improvement, fell within ±MID, or met the MID for deterioration (MID ≈ 10 points for EORTC QLQ-C30 scales; 1.3 for anxiety and 1.4 for depression).

**Table 4:** Node Centrality at Admission (T0) and Discharge (T1).

Node	Domain	EI T0	EI T1	Bridge EI T0	95% CI	Bridge EI T1	95% CI
EF	Emotional functioning	−0.833	−0.756	1.103	[1.060, 1.196]	1.129	[1.065, 1.206]
ANX	Anxiety (HADS)	+0.193	+0.244	1.052	[1.002, 1.177]	0.951	[0.886, 1.074]
PF	Physical functioning	−0.467	−0.434	1.051	[0.973, 1.137]	0.985	[0.912, 1.076]
FA	Fatigue	−0.526	−0.306	1.10	[1.037, 1.162]	0.920	[0.868, 0.993]
DEP	Depression (HADS)	−0.265	−0.334	0.864	[0.797, 0.989]	0.845	[0.777, 0.973]
PA	Pain	−0.148	−0.176	0.698	[0.627, 0.785]	0.718	[0.648, 0.802]
CF	Cognitive functioning	−0.330	−0.257	0.643	[0.573, 0.733]	0.587	[0.534, 0.678]
GH	Global health status	−0.201	−0.143	0.631	[0.584, 0.728]	0.673	[0.630, 0.789]
SF	Social functioning	+0.416	+0.169	0.559	[0.485, 0.661]	0.573	[0.503, 0.679]
RF	Role functioning	+0.253	+0.266	0.535	[0.503, 0.656]	0.572	[0.514, 0.683]
FI	Financial impact	−0.285	−0.367	0.425	[0.388, 0.514]	0.569	[0.507, 0.648]
SL	Insomnia	+0.425	+0.537	0.467	[0.409, 0.539]	0.442	[0.399, 0.509]
DY	Dyspnoea	−0.067	−0.043	0.384	[0.334, 0.479]	0.390	[0.334, 0.487]
AP	Appetite loss	+0.515	+0.262	0.332	[0.272, 0.414]	0.271	[0.233, 0.346]
DI	Diarrhea	+0.081	+0.016	0.190	[0.138, 0.276]	0.194	[0.152, 0.282]
CO	Constipation	+0.077	+0.081	0.168	[0.139, 0.263]	0.195	[0.160, 0.297]
NV	Nausea/vomiting	+0.798	+0.639	0.177	[0.132, 0.283]	0.150	[0.110, 0.253]

Note. EI = Expected Influence (signed sum of BMA-weighted partial correlations). Bridge EI = Bridge Expected Influence across three communities (Functioning, Symptoms, Psychological). Nodes ordered by Bridge EI T0 descending. Causal directionality cannot be inferred. **Abbreviations**: **PF**: physical functioning; **RF**: role functioning; **SF**: social functioning; **EF**: emotional functioning; **CF**: cognitive functioning; **GH**: global health status; **FA**: fatigue; **NV**: nausea/vomiting; **PA**: pain; **DY**: dyspnoea; **SL**: insomnia; **AP**: appetite loss; **CO**: constipation; **DI**: diarrhea; **FI**: financial impact; **ANX**: HADS anxiety; **DEP**: HADS depression.

## Data Availability

The research data supporting this publication are stored in our institutional digital data repository for published research, accessible via https://creed.lbg.ac.at. The datasets analyzed in this manuscript are not publicly available due to ethical and legal restrictions, as they contain potentially identifying and sensitive patient information. However, pseudonymized datasets have been created for the purpose of re-use and are also accessible at creed.lbg.ac.at. Requests for access to anonymized datasets should be directed to the corresponding author (D.R.).

## Supplementary Materials

The following supporting information can be downloaded at: https://www.mdpi.com/article/10.3390/cancers18132155/s1, Table S1: BMA-Weighted Partial Correlations for All 136 Edges at Admission (T0) and Discharge (T1).
