# Supplementary material for: Changes in Symptom Networks During Inpatient Cancer Rehabilitation: A Retrospective Bayesian Gaussian Graphical Model Analysis of Real-World Patient-Reported Outcomes
Source: Cancers (Basel). 2026 Jul 4;18(13):2155. doi: 10.3390/cancers18132155 (PMC13359449; doi:10.3390/cancers18132155)
Supplement: Supplementary file 1 [file cancers-18-02155-s001.zip › cancers-4363664-supplementary.pdf]

cancers

MDPI

Changes in Symptom Networks During Inpatient Cancer Rehabilitation: A Retrospective Bayesian Network Analysis Based on Real World Data

Kirchhoff, C. [1], Licht, T. [2,3] *, Eke, S. [1], Matko, S. [2], Grote, V. [2], Fischer, M.J. [2,4], Hüfner, K. [1], Riedl and D. [1,2] *

BMA-Weighted Partial Correlations for All 136 Edges at Admission (T0) and Discharge (T1)

Table S1 provides the numerical edge weights and change statistics for all 136 unique edges of the 17-node network. BMA-weighted partial correlations represent the posterior expectation of each pairwise partial correlation, averaged across all candidate network structures weighted by their posterior probability. Positive values indicate that two nodes are positively associated conditional on all other nodes in the network; negative values indicate inverse associations. For each edge, the table reports the partial-correlation weight and posterior inclusion probability at admission (T0) and discharge (T1), the posterior mean difference (Δ = T0 − T1) with its 95% Highest Density Interval, the posterior probability of direction, whether the 99% HDI excluded zero, and the region-of-practical-equivalence (ROPE) classification. Highlighted rows (n = 23) indicate edges whose 95% HDI excluded zero (credible change); these are listed first, ordered by absolute Δ, followed by the remaining edges.

Table S1. BMA-Weighted Partial Correlations for All 136 Edges at Admission (T0) and Discharge (T1).

Node 1	Node 2	PIP T0	r T0	PIP T1	r T1	Δ (T0–T1)	95% HDI	pd	99% HDI≠0	ROPE
**Social functioning**	**Financial impact**	1.00	−0.164	1.00	−0.276	**−0.112**	[−0.149, −0.076]	1.000	✓	Outside
**Role functioning**	**Emotional functioning**	1.00	−0.059	0.16	0.023	**+0.083**	[0.043, 0.120]	1.000	✓	Undecided
**Nausea/vomiting**	**Anxiety**	0.99	0.054	0.15	−0.022	**−0.076**	[−0.115, −0.038]	1.000	✓	Undecided
**Cognitive functioning**	**Financial impact**	1.00	−0.119	0.99	−0.054	**+0.065**	[0.027, 0.105]	1.000	✓	Undecided
**Role functioning**	**Fatigue**	1.00	−0.196	1.00	−0.132	**+0.064**	[0.026, 0.102]	1.000	✓	Undecided
**Cognitive functioning**	**Depression**	1.00	−0.061	1.00	−0.119	**−0.058**	[−0.098, −0.020]	0.998	✓	Undecided
**Physical functioning**	**Anxiety**	1.00	0.076	0.10	0.018	**−0.058**	[−0.097, −0.019]	0.998	✓	Undecided
**Fatigue**	**Anxiety**	1.00	−0.109	0.98	−0.052	**+0.058**	[0.018, 0.096]	0.998	✓	Undecided
**Social functioning**	**Global QoL**	1.00	0.133	1.00	0.078	**−0.055**	[−0.094, −0.017]	0.998	✓	Undecided
**Pain**	**Constipation**	1.00	0.063	0.05	0.008	**−0.055**	[−0.093, −0.016]	0.997	✓	Undecided
**Appetite loss**	**Diarrhoea**	1.00	0.084	0.36	0.031	**−0.053**	[−0.091, −0.015]	0.997	✓	Undecided

Node 1	Node 2	PIP T0	r T0	PIP T1	r T1	Δ (T0–T1)	95% HDI	pd	99% HDI≠0	ROPE
**Nausea/vomiting**	**Appetite loss**	1.00	0.350	1.00	0.297	**−0.053**	[−0.088, −0.019]	0.998	✓	Undecided
**Social functioning**	**Depression**	1.00	−0.093	0.77	−0.040	**+0.053**	[0.013, 0.091]	0.996	✓	Undecided
**Anxiety**	**Depression**	1.00	0.443	1.00	0.391	**−0.052**	[−0.084, −0.019]	0.999	✓	Undecided
**Emotional functioning**	**Fatigue**	1.00	−0.277	1.00	−0.230	**+0.048**	[0.012, 0.084]	0.994	—	Undecided
**Global QoL**	**Financial impact**	0.14	−0.022	0.18	0.024	**+0.046**	[0.007, 0.084]	0.991	—	Undecided
**Emotional functioning**	**Appetite loss**	0.06	−0.010	0.99	−0.056	**−0.046**	[−0.084, −0.007]	0.990	—	Undecided
**Fatigue**	**Nausea/vomiting**	1.00	0.089	0.87	0.044	**−0.045**	[−0.084, −0.006]	0.991	—	Undecided
**Role functioning**	**Social functioning**	1.00	0.246	1.00	0.204	**−0.042**	[−0.079, −0.005]	0.987	—	Undecided
**Physical functioning**	**Financial impact**	0.76	−0.040	1.00	−0.080	**−0.041**	[−0.080, −0.002]	0.979	—	Undecided
**Role functioning**	**Appetite loss**	0.05	0.006	0.49	−0.034	**−0.040**	[−0.080, −0.003]	0.979	—	Undecided
**Emotional functioning**	**Nausea/vomiting**	0.05	−0.001	0.71	−0.039	**−0.038**	[−0.078, −0.000]	0.974	—	Undecided
**Emotional functioning**	**Anxiety**	1.00	−0.432	1.00	−0.397	**+0.035**	[0.000, 0.065]	0.983	—	Undecided
Appetite loss	Anxiety	0.93	−0.047	0.05	−0.007	+0.040	[−0.000, 0.077]	0.977	—	Undecided
Cognitive functioning	Appetite loss	0.53	0.035	0.05	−0.005	−0.040	[−0.078, 0.000]	0.975	—	Undecided
Appetite loss	Depression	1.00	0.078	0.77	0.041	−0.038	[−0.076, 0.001]	0.973	—	Undecided
Physical functioning	Appetite loss	0.96	−0.050	0.08	−0.015	+0.035	[−0.004, 0.074]	0.962	—	Undecided
Social functioning	Constipation	0.12	−0.020	0.06	0.011	+0.031	[−0.007, 0.070]	0.944	—	Undecided
Global QoL	Pain	1.00	−0.128	1.00	−0.159	−0.031	[−0.068, 0.008]	0.941	—	Undecided
Fatigue	Diarrhoea	0.05	0.007	0.66	0.038	+0.031	[−0.009, 0.068]	0.939	—	Undecided
Physical functioning	Role functioning	1.00	0.255	1.00	0.285	+0.031	[−0.004, 0.067]	0.954	—	Undecided
Physical functioning	Social functioning	0.74	0.040	0.06	0.009	−0.030	[−0.071, 0.007]	0.937	—	Undecided
Role functioning	Anxiety	0.61	0.037	1.00	0.067	+0.030	[−0.009, 0.068]	0.936	—	Undecided
Cognitive functioning	Fatigue	1.00	−0.084	1.00	−0.114	−0.030	[−0.069, 0.008]	0.936	—	Undecided
Physical functioning	Global QoL	1.00	0.130	1.00	0.160	+0.029	[−0.008, 0.068]	0.935	—	Undecided
Global QoL	Anxiety	0.06	0.011	0.76	0.040	+0.029	[−0.008, 0.069]	0.931	—	Undecided
Dyspnoea	Constipation	0.05	0.000	0.30	−0.029	−0.029	[−0.068, 0.008]	0.928	—	Undecided
Fatigue	Insomnia	1.00	0.113	1.00	0.141	+0.029	[−0.009, 0.067]	0.928	—	Undecided
Pain	Dyspnoea	0.14	−0.022	0.05	0.005	+0.027	[−0.010, 0.067]	0.920	—	Undecided
Insomnia	Depression	0.07	−0.014	0.07	0.013	+0.027	[−0.013, 0.066]	0.908	—	Undecided

Node 1	Node 2	PIP T0	r T0	PIP T1	r T1	Δ (T0–T1)	95% HDI	pd	99% HDI≠0	ROPE
Appetite loss	Financial impact	0.05	0.003	0.16	−0.023	−0.026	[−0.064, 0.013]	0.911	—	Undecided
Cognitive functioning	Global QoL	0.64	−0.037	0.06	−0.011	+0.026	[−0.013, 0.065]	0.904	—	Undecided
Physical functioning	Diarrhoea	0.10	−0.018	0.05	0.008	+0.025	[−0.014, 0.065]	0.901	—	Undecided
Role functioning	Global QoL	1.00	0.092	1.00	0.067	−0.025	[−0.064, 0.013]	0.902	—	Undecided
Social functioning	Emotional functioning	1.00	0.167	1.00	0.142	−0.025	[−0.062, 0.013]	0.900	—	Undecided
Cognitive functioning	Insomnia	1.00	−0.086	1.00	−0.062	+0.025	[−0.013, 0.064]	0.893	—	Undecided
Insomnia	Diarrhoea	0.33	0.030	0.05	0.006	−0.024	[−0.064, 0.014]	0.889	—	Undecided
Cognitive functioning	Pain	0.07	−0.012	0.06	0.012	+0.024	[−0.015, 0.064]	0.891	—	Undecided
Constipation	Anxiety	0.05	0.001	0.16	−0.023	−0.024	[−0.064, 0.014]	0.891	—	Undecided
Pain	Anxiety	0.33	0.030	0.99	0.054	+0.024	[−0.015, 0.063]	0.887	—	Undecided
Constipation	Diarrhoea	1.00	−0.071	0.94	−0.047	+0.024	[−0.016, 0.062]	0.879	—	Undecided
Fatigue	Dyspnoea	1.00	0.157	1.00	0.180	+0.023	[−0.015, 0.061]	0.893	—	Undecided
Social functioning	Pain	0.99	0.053	0.35	0.030	−0.022	[−0.060, 0.017]	0.872	—	Undecided
Dyspnoea	Anxiety	0.06	0.009	0.38	0.031	+0.022	[−0.015, 0.063]	0.864	—	Undecided
Physical functioning	Fatigue	1.00	−0.249	1.00	−0.227	+0.022	[−0.014, 0.059]	0.883	—	Undecided
Global QoL	Insomnia	0.07	−0.014	0.06	0.008	+0.022	[−0.017, 0.061]	0.869	—	Undecided
Social functioning	Anxiety	0.97	0.050	0.29	0.028	−0.022	[−0.061, 0.016]	0.867	—	Undecided
Fatigue	Appetite loss	1.00	0.108	1.00	0.086	−0.022	[−0.059, 0.018]	0.866	—	Undecided
Pain	Diarrhoea	0.05	0.003	0.10	−0.018	−0.022	[−0.060, 0.017]	0.864	—	Undecided
Role functioning	Pain	1.00	−0.171	1.00	−0.191	−0.020	[−0.058, 0.018]	0.851	—	Undecided
Global QoL	Depression	1.00	−0.204	1.00	−0.224	−0.020	[−0.057, 0.017]	0.854	—	Undecided
Emotional functioning	Insomnia	1.00	−0.105	1.00	−0.086	+0.019	[−0.019, 0.057]	0.835	—	Undecided
Nausea/vomiting	Pain	0.99	0.055	1.00	0.073	+0.019	[−0.020, 0.058]	0.828	—	Undecided
Role functioning	Diarrhoea	0.05	0.006	0.07	−0.012	−0.019	[−0.058, 0.019]	0.823	—	Undecided
Emotional functioning	Constipation	0.11	−0.019	0.64	−0.037	−0.019	[−0.057, 0.021]	0.825	—	Undecided
Social functioning	Diarrhoea	0.95	−0.048	1.00	−0.066	−0.018	[−0.058, 0.019]	0.822	—	Undecided
Role functioning	Nausea/vomiting	0.34	0.030	0.07	0.012	−0.018	[−0.056, 0.022]	0.814	—	Undecided
Financial impact	Depression	0.05	−0.007	0.18	−0.024	−0.018	[−0.057, 0.022]	0.809	—	Undecided
Fatigue	Constipation	0.19	0.024	0.82	0.042	+0.018	[−0.023, 0.055]	0.815	—	Undecided
Role functioning	Depression	0.05	−0.002	0.12	−0.020	−0.017	[−0.056, 0.022]	0.809	—	Undecided

Node 1	Node 2	PIP T0	r T0	PIP T1	r T1	Δ (T0–T1)	95% HDI	pd	99% HDI≠0	ROPE
Constipation	Depression	0.06	−0.011	0.05	0.006	+0.017	[−0.021, 0.057]	0.799	—	Undecided
Emotional functioning	Financial impact	1.00	−0.058	1.00	−0.074	−0.017	[−0.055, 0.023]	0.799	—	Undecided
Financial impact	Anxiety	0.07	0.014	0.33	0.030	+0.017	[−0.023, 0.055]	0.796	—	Undecided
Social functioning	Cognitive functioning	1.00	0.083	1.00	0.099	+0.016	[−0.022, 0.056]	0.800	—	Undecided
Emotional functioning	Cognitive functioning	1.00	0.186	1.00	0.201	+0.016	[−0.021, 0.054]	0.794	—	Undecided
Global QoL	Fatigue	1.00	−0.123	1.00	−0.108	+0.015	[−0.022, 0.053]	0.780	—	Undecided
Dyspnoea	Financial impact	0.10	0.018	0.05	0.003	−0.015	[−0.053, 0.024]	0.769	—	Undecided
Global QoL	Constipation	0.06	−0.009	0.05	0.005	+0.015	[−0.024, 0.054]	0.767	—	Undecided
Global QoL	Nausea/vomiting	0.07	−0.013	0.05	0.002	+0.015	[−0.025, 0.053]	0.765	—	Undecided
Emotional functioning	Pain	1.00	−0.079	1.00	−0.064	+0.014	[−0.024, 0.053]	0.766	—	Undecided
Physical functioning	Cognitive functioning	0.06	−0.009	0.05	0.005	+0.014	[−0.023, 0.054]	0.767	—	Undecided
Diarrhoea	Financial impact	0.06	0.009	0.05	−0.004	−0.014	[−0.053, 0.024]	0.762	—	Undecided
Nausea/vomiting	Depression	0.59	−0.036	0.15	−0.022	+0.014	[−0.025, 0.052]	0.760	—	Undecided
Insomnia	Anxiety	1.00	0.120	1.00	0.134	+0.014	[−0.026, 0.051]	0.767	—	Undecided
Emotional functioning	Dyspnoea	0.05	0.007	0.13	0.020	+0.014	[−0.024, 0.052]	0.759	—	Undecided
Physical functioning	Constipation	0.16	−0.023	0.60	−0.037	−0.014	[−0.052, 0.026]	0.756	—	Undecided
Dyspnoea	Appetite loss	0.10	−0.018	0.05	−0.005	+0.013	[−0.026, 0.051]	0.752	—	Undecided
Nausea/vomiting	Constipation	1.00	0.105	1.00	0.118	+0.013	[−0.025, 0.052]	0.748	—	Undecided
Social functioning	Dyspnoea	0.15	0.022	0.06	0.009	−0.013	[−0.053, 0.024]	0.742	—	Undecided
Insomnia	Appetite loss	0.33	0.030	0.10	0.018	−0.013	[−0.052, 0.025]	0.739	—	Undecided
Nausea/vomiting	Insomnia	0.05	−0.008	0.05	0.005	+0.012	[−0.025, 0.052]	0.730	—	Undecided
Pain	Depression	1.00	−0.065	0.99	−0.053	+0.012	[−0.026, 0.052]	0.723	—	Undecided
Physical functioning	Nausea/vomiting	0.33	−0.030	0.81	−0.042	−0.012	[−0.050, 0.027]	0.725	—	Undecided
Cognitive functioning	Dyspnoea	1.00	−0.069	1.00	−0.059	+0.011	[−0.028, 0.050]	0.715	—	Within
Global QoL	Diarrhoea	0.31	−0.029	0.11	−0.019	+0.011	[−0.028, 0.050]	0.708	—	Within
Pain	Appetite loss	0.41	−0.032	0.85	−0.043	−0.011	[−0.049, 0.029]	0.709	—	Within
Role functioning	Cognitive functioning	0.12	0.021	0.06	0.011	−0.010	[−0.050, 0.028]	0.695	—	Within
Dyspnoea	Insomnia	1.00	0.059	0.96	0.049	−0.010	[−0.048, 0.030]	0.691	—	Within
Global QoL	Appetite loss	1.00	−0.071	1.00	−0.081	−0.010	[−0.048, 0.029]	0.691	—	Within
Physical functioning	Insomnia	1.00	0.066	1.00	0.075	+0.009	[−0.030, 0.049]	0.687	—	Within
Constipation	Financial impact	0.07	−0.014	0.05	−0.004	+0.009	[−0.030, 0.048]	0.673	—	Within

Node 1	Node 2	PIP T0	r T0	PIP T1	r T1	Δ (T0–T1)	95% HDI	pd	99% HDI≠0	ROPE
Role functioning	Insomnia	0.58	0.036	0.89	0.044	+0.009	[−0.030, 0.047]	0.677	—	Within
Global QoL	Dyspnoea	0.05	0.005	0.05	−0.004	−0.009	[−0.047, 0.030]	0.678	—	Within
Cognitive functioning	Anxiety	1.00	−0.057	0.95	−0.048	+0.009	[−0.031, 0.047]	0.671	—	Within
Fatigue	Financial impact	0.64	−0.037	0.28	−0.028	+0.009	[−0.032, 0.047]	0.669	—	Within
Social functioning	Fatigue	0.91	−0.045	0.99	−0.054	−0.009	[−0.046, 0.031]	0.670	—	Within
Diarrhoea	Depression	0.46	−0.033	0.19	−0.025	+0.009	[−0.029, 0.048]	0.673	—	Within
Cognitive functioning	Diarrhoea	0.25	−0.027	0.55	−0.036	−0.008	[−0.048, 0.030]	0.657	—	Within
Nausea/vomiting	Financial impact	0.24	0.027	0.55	0.035	+0.008	[−0.031, 0.048]	0.658	—	Within
Fatigue	Pain	1.00	0.103	1.00	0.111	+0.008	[−0.031, 0.046]	0.662	—	Within
Physical functioning	Pain	1.00	−0.161	1.00	−0.154	+0.008	[−0.030, 0.046]	0.665	—	Within
Cognitive functioning	Nausea/vomiting	0.06	−0.010	0.05	−0.002	+0.008	[−0.032, 0.047]	0.651	—	Within
Diarrhoea	Anxiety	0.05	−0.006	0.05	0.001	+0.007	[−0.032, 0.046]	0.630	—	Within
Pain	Insomnia	1.00	0.116	1.00	0.109	−0.007	[−0.045, 0.030]	0.635	—	Within
Dyspnoea	Diarrhoea	0.20	0.025	0.11	0.019	−0.006	[−0.046, 0.032]	0.630	—	Within
Insomnia	Constipation	0.94	0.048	0.99	0.054	+0.006	[−0.031, 0.046]	0.622	—	Within
Pain	Financial impact	1.00	0.099	1.00	0.104	+0.006	[−0.033, 0.044]	0.618	—	Within
Emotional functioning	Depression	1.00	−0.093	1.00	−0.098	−0.006	[−0.045, 0.032]	0.611	—	Within
Social functioning	Insomnia	0.19	0.025	0.11	0.019	−0.006	[−0.043, 0.034]	0.611	—	Within
Appetite loss	Constipation	1.00	0.084	1.00	0.090	+0.005	[−0.033, 0.044]	0.607	—	Within
Emotional functioning	Diarrhoea	0.16	−0.023	0.28	−0.028	−0.005	[−0.044, 0.033]	0.607	—	Within
Nausea/vomiting	Dyspnoea	0.06	0.011	0.05	0.005	−0.005	[−0.044, 0.034]	0.607	—	Within
Social functioning	Nausea/vomiting	0.05	0.003	0.05	0.008	+0.005	[−0.034, 0.044]	0.603	—	Within
Fatigue	Depression	0.05	−0.008	0.05	−0.003	+0.005	[−0.034, 0.044]	0.601	—	Within
Physical functioning	Dyspnoea	1.00	−0.200	1.00	−0.195	+0.005	[−0.031, 0.043]	0.602	—	Within
Cognitive functioning	Constipation	1.00	−0.082	1.00	−0.076	+0.005	[−0.033, 0.044]	0.595	—	Within
Role functioning	Financial impact	0.05	−0.002	0.05	−0.007	−0.005	[−0.044, 0.034]	0.593	—	Within
Role functioning	Dyspnoea	0.96	−0.048	0.98	−0.052	−0.004	[−0.043, 0.035]	0.589	—	Within
Nausea/vomiting	Diarrhoea	1.00	0.171	1.00	0.167	−0.004	[−0.042, 0.033]	0.577	—	Within
Physical functioning	Emotional functioning	1.00	−0.116	1.00	−0.111	+0.004	[−0.034, 0.043]	0.572	—	Within

Node 1	Node 2	PIP T0	r T0	PIP T1	r T1	Δ (T0–T1)	95% HDI	pd	99% HDI≠0	ROPE
Social functioning	Appetite loss	0.55	−0.035	0.38	−0.032	+0.004	[−0.035, 0.042]	0.578	—	Within
Physical functioning	Depression	1.00	−0.138	1.00	−0.135	+0.004	[−0.034, 0.042]	0.571	—	Within
Emotional functioning	Global QoL	1.00	0.078	1.00	0.078	+0.001	[−0.037, 0.040]	0.525	—	Within
Role functioning	Constipation	0.05	0.002	0.05	0.000	−0.001	[−0.041, 0.037]	0.525	—	Within
Dyspnoea	Depression	0.15	−0.022	0.13	−0.021	+0.001	[−0.039, 0.039]	0.521	—	Within
Insomnia	Financial impact	0.06	0.009	0.06	0.010	+0.001	[−0.038, 0.040]	0.517	—	Within

Note. PIP = Posterior Inclusion Probability (posterior probability that an edge is present, range 0–1). r = Bayesian Model Averaging–weighted partial correlation (dimensionless, range −1 to +1); positive values indicate positive association, negative values inverse association, conditional on all other nodes. Δ = posterior mean difference in partial-correlation weight (T0 − T1). The sign of Δ reflects the direction of the numerical difference only; because edges may be positive or negative, a given sign does not by itself indicate strengthening or weakening (see Table 3 for the strength interpretation of credibly changed edges, based on the change in absolute weight). pd = posterior probability of direction. "99% HDI≠0" indicates whether the 99% Highest Density Interval of the difference excluded zero. ROPE (±0.05): "Outside" = 95% HDI entirely outside the region of practical equivalence (non-trivial change); "Within" = 95% HDI entirely within it (positive evidence of practical equivalence); "Undecided" = neither. Of the 23 credibly changed edges, 14 also excluded zero at the 99% HDI and 1 lay outside the ROPE; 43 of all 136 edges fell within the ROPE. All edge-level comparisons are exploratory. Highlighted rows (n = 23) indicate credible change. Edges are sorted with credibly changed edges first, then by descending |Δ|.
